# Volatiles extracted from *Melaleuca Rugulosa* (Link) Craven leaves: comparative profiling, bioactivity screening, and metabolomic analysis

**DOI:** 10.1186/s12906-024-04683-z

**Published:** 2024-11-13

**Authors:** Mohamed S. Mady, Heba E. Elsayed, Nashwa F Tawfik, Fatma A. Moharram

**Affiliations:** Department of Pharmacognosy, Faculty of Pharmacy, Helwan University, Cairo, 11795 Egypt

**Keywords:** Antibacterial, Anti-aging, Essential oil, *Melaleuca Rugulosa*, Metabolomics

## Abstract

**Background:**

Melaleuca species (family Myrtaceae) are characterized by their wide-ranging applications as antimicrobials and in skin-related conditions. Herein, we estimated the volatile profile and biological significance of *M. rugulosa* (Link) leaves for the first time supported by a dereplication protocol.

**Materials and methods:**

Volatile components were extracted using hydrodistillation (HD), supercritical fluid (SF), and headspace (HS) techniques and identified using GC/MS. The variations among the three extracts were assessed using principal component analysis and orthogonal partial least square discriminant analysis (OPLS-DA). The extracted volatiles were tested for radical scavenging activity, anti-aging, and anti-hyperpigmentation potential. Finally, disc diffusion and broth microdilution assays were implemented to explore the antibacterial capacity against *Streptococcus pyogenes*,* Staphylococcus aureus*,* Clostridium perfringens*, and *Pseudomonas aeruginosa.*

**Results:**

The yield of the SF technique (0.8%) was three times higher than HD. GC/MS analysis revealed that the oxygenated compounds are the most proponents in the three extracts being 95.93% (HD), 80.94% (HS), and 48.4% (SF). Moreover, eucalyptol (1,8-cineol) represents the major component in the HD-EO (89.60%) and HS (73.13%) volatiles, while *dl*-*α*-tocopherol (16.27%) and *α*-terpineol (11.89%) represent the highest percentage in SF extract. Regarding the bioactivity profile, the HD-EO and SF-extract showed antioxidant potential in terms of oxygen radical absorbance capacity, and *β*- carotene assays, while exerting weak activity towards DPPH. In addition, they displayed potent anti-elastase and moderate anti-collagenase activities. The HD-EO exhibited potent anti-tyrosinase activity, while the SF extract showed a moderate level compared to tested controls. OPLS-DA and dereplication studies predicted that the selective antibacterial activity of HD-EO to *S. aureus* was related to eucalyptol, while SF extract to *C. perfringens* was related to *α*-tocopherol.

**Conclusions:**

*M. rugulosa* leaves are considered a vital source of bioactive volatile components that are promoted for controlling skin aging and infection. However, further safety and clinical studies are recommended.

**Supplementary Information:**

The online version contains supplementary material available at 10.1186/s12906-024-04683-z.

## Introduction

Skin aging is considered a challenging, multifactorial process [1]. It involves secondary structural changes of the skin, underlying muscles, and subcutaneous fat tissue [1] owing to different exogenous and indigenous factors. Exogenous factors are mostly due to environmental issues, such as smoking, pollution, and excessive exposure to UV light [2, 3] which encourages the excessive production of reactive oxygen species (ROS) that leads to oxidative stress in the tissue and stimulates extracellular matrix (ECM) degradation. The degradation of ECM is attributed to skin aging through the activation of collagenase, elastase, and tyrosinase as the main skin aging enzymes. Subsequent activation of these enzymes causes a significant reduction in the collagen and elastin levels, resulting in the loss of the elasticity and strength of the skin, in addition to wrinkle formation [4]. Concurrently, tyrosinase is the rate-limiting enzyme in melanin formation, and its overproduction results in melanin accumulation, skin tanning, and hyperpigmentation. Hence, among other natural-based treatments, antioxidants are promoted as the first line for controlling skin aging as they possess excellent defense mechanisms by scavenging ROS [5]. Moreover, elastase, collagenase, and tyrosinase inhibitors endorse skin elasticity and reduce hyperpigmentation complaints [6]. So, searching for natural products-based remedies with the aforementioned activities is a pragmatic plan.

Microbial flora is intimately associated with the human skin and mucous membranes from birth until death. This relationship offers protection against infections by pathogenic species *via* surface competition and the production of antimicrobial substances [7, 8]. The latter results in cross-reactive antibodies which are active against other aggressive microbes. Some factors such as temperature, moisture, and poor hygiene may cause an imbalance between the host and microbial flora by exaggerating the overgrowth of exogenous bacteria, and fungi causing skin infection [7, 9]. Routinely, topical antimicrobial agents are used to manage skin infections, but the emergence of drug-resistant bacteria such as *Pseudomonas aeruginosa*, and *Staphylococcus aureus* is a major challenge. Additionally, other bacteria are considered a source of potentially serious skin infections with costly and limited treatment options such as *Clostridium perfringens* (causative agent for gas gangrene) and *Streptococcus pyogenes* that trigger skin and soft tissue infections (such as impetigo and cellulitis) [10–12]. Therefore, there is a rising request to search for a suitable treatment for skin infections to overcome the resistance, side effects, and high cost of synthetic drugs.

Essential oil (EO) is a unique class of natural products that possesses a hydrophobic, complex chemical nature with various pharmacological activities [13–19]. EOs are ubiquitous in the genera of the family Myrtaceae especially Melaleuca [20]. Genus Melaleuca includes about 290 species ranging from small shrubs and trees, it is native to Australia and commonly known as tea tree, while some species are natively grown in India [21]. Traditionally, Melaleuca species were used to cure various ailments, while several research groups have addressed their therapeutic potential in several pharmacological settings [22–27]. *Melaleuca rugulosa* (Link) Craven (Synonym *Callistemon macropunctatus*) is native to South Australia and cultivated worldwide as an ornamental tree [25]. Its EO is considered an alternative source for tea tree oil and is renowned for its therapeutic benefits in the management of acne, inflammation of the skin [27], and microbial infections [28, 29]. Conversely, there is little information about the EOs of *M. rugulosa* except for the antibacterial and anticancer activities of the species cultivated in Australia and India [23, 27]. Based on the reported literature, and in continuation of our work on the EOs extracted from various Melaleuca species [26, 28], this study intended to compare, for the first time, the extracted volatile constituents from the *M. rugulosa* leaves, cultivated in Egypt, by three different techniques. In addition, antioxidant, antiaging, anti-hyperpigmentation, and antibacterial activities were evaluated. Ultimately a metabolomic study was conducted, in an attempt, to correlate the observed bioactivity to the identified volatile constituents.

## Materials and methods

### Plant material

Fresh leaves were collected around the flowering growth stage of an ornamental *M. rugulosa* (Link) Craven tree from El-Orman Garden with the coordinates 30°01′45″N 31°12′47″E (El-Giza, Egypt; March 2023) according to the guidelines endorsed by the local garden`s and following the scientific collection guidelines of Egypt. It was identified by Dr. Trease Labib, a taxonomy specialist at Mazhar Garden, (Giza, Egypt; ) and preserved at the official Herbarium of the Faculty of Pharmacy, Department of Pharmacognosy, Helwan University (Cairo, Egypt; 02 Mru/2018). Another sample was deposited at El Orman Public Botanical Garden`s Herbarium (El Giza-Egypt) with voucher sample number 001150MR-000118-04-07-04-00118.

### Chemicals and reagents

2,2-Diphenyl-1-picrylhydrazyl (DPPH), ascorbic acid, Trolox, phosphate buffer, butylhydroxytoluene (BHT, standard), Tween 20, linoleic acid, β-carotene, methoxy succinyl-alanyl-alanyl-prolyl-valine chloromethyl ketone (MeOSuc-AAPV-CMK), 1.0 mM of N-methoxy succinyl - Ala - Ala - Pro -Val - *p* -nitroanilide, FALGPA substrate (N-(3-[2-Furyl] acryloyl)-Leu-Gly-Pro-Ala, ninhydrin, citrate buffer, kojic acid, L-DOPA and tricine buffer were purchased from Sigma-Aldrich, United States.

### Extraction of volatile constituents

#### Hydro-distillation (HD) method

*M. rugulosa* fresh leaves (600 g) were extracted using the conventional hydro distillation technique according to Elsayed et al. [30]. The plant material was mixed with distilled water and extracted by Clevenger apparatus (4 h). The obtained oil was dehydrated using anhydrous sodium sulfate to remove water droplets.

#### Supercritical fluid (SF) extraction

Dry leaves (500 g) were subjected to supercritical fluid (SF) extraction using CO_2_ in supercritical conditions according to Elsayed et al. [30], following the procedure of Rodriguez et al. [31]. USDA1-apparatus accompanied by supercritical CO_2_ and ethanol as co-solvent was used for the separation process at 40 °C and the flow rate was 10 mL/min for 1 h static and dynamic mode respectively. The percentage of the HD and SF EOs was stated in mL / 100 g of plant material, and they were stored in the refrigerator (4 °C) till GC/MS analysis.

#### Head-space (HS)

The HS micro-extraction of the volatile constituents from *M. rugulosa* fresh leaves was performed according to Rodriguez et al. [31] and Elsayed et al. [30]. About 1 g fresh leaf, was placed in a Shimadzu headspace sampler HS-20 glass vial (5 mL, 60–70 °C) coupled to a Shimadzu GCMS-QP2020 GC/MS (Koyoto, Japan) equipped with Rtx-1MS column (30 m × 0.25 mm id. × 0.25 μm film thickness) (Restek, Bellefonte, PA, United States). The analysis of the volatile constituents in the three methods was carried out following the condition stated in the supplementary data.

### Identification of essential oil components

The acquired volatile constituents from the three extracted EOs were analyzed in triplicate and the mean was calculated. The tentative identification of the oil components was implemented by comparing their Kovats’ retention index (RI) with *the n*-alkanes standard (C_8_-C_28_). In addition, the obtained mass spectra were matched with that reported in the National Institute of Standards and Technology (NIST) and mass spectral database (Wiley, in addition to comparison with published data (similarity index > 90%) [32].

### Mass spectral data processing and metabolite profiling

The mass spectral data process was done using MZmine 2.53 software (http://mzmine.sourceforge.net/) and the peaks were detected using the chromatogram builder. Mass detection was performed using the exact mass detector with a noise level set at 500. The chromatogram builder used the highest data point function. The minimum time was set at 2.0 s, and the minimum height and *m/z* tolerance were 500 and 0.001, respectively. Chromatogram deconvolution was then performed to detect the individual peaks [33]. Finally, the peak lists of the EOs were aligned using the join aligner parameters set at minimum confidence 0.1 retention time tolerance 0.1 *m/z* tolerance: 0.001, score weight 0.5. The data were then exported as a CSV file for further processing which was then imported into SIMCA-P V 14.1 (Umetrics, Umeå, Sweden) [34].

### In vitro antioxidant activity

#### 2,2-Diphenyl-1-picrylhydrazyl (DPPH) radical scavenging assay

It was performed according to the method stated earlier by Ebrahim et al. [35]. Briefly, 100 µL of DPPH in methanol was added to an equivalent amount of different concentrations of oil samples and ascorbic acid (as a standard). The mixture was left for 30 min in the dark, then the absorbances were detected at λ_517_. The antioxidant activity was calculated as a percentage of DPPH inhibition (I%) by the following equation: [(Control absorbance – Sample absorbance /control absorbance] x 100]

#### Oxygen radical absorbance capacity (ORAC)

It was implemented according to Ebrahim et al. [35]. Different concentrations of EOs were added to 10 mM, phosphate buffer (pH 7.4), and fluorescein dye. The main mechanism to measure the antioxidant potential is based on detecting the time required by the fluorescein dye to lose its fluorescence compared to Trolox (Standard).

#### β-Carotene bleaching test

Lipid peroxidation inhibition was evaluated according to Ebrahim et al. [35]. Briefly, different concentrations of the EOs, and butylhydroxytoluene (BHT, standard) were added to a mixture of Tween 20, linoleic acid, and β-carotene then the absorbance was measured at λ_max_ 470 nm at zero, and after 1.0 h. The inhibition percent was calculated according to the given equation: I_bleaching_ (%) = 1- (A_0−_ A_t_ / A^0^_0−_ A^0^_t_ )x 100.

where A_0_ and A^0^_0_ are the samples or standard and control absorbance, respectively at zero time, while A _t_ and A^0^_t_ are the sample or standard and control, respectively after one hour.

In the three methods, the experiments were performed in triplicates and the nonlinear regression analysis was used to obtain the IC_50_ using curves constructed between the measured absorbance and concentration.

### Evaluation of anti-elastase, anti-collagenase, and anti-tyrosinase activity

They were evaluated according to the procedure mentioned by Mostafa et al., 2021 [36].

#### Estimation of anti-elastase activity

Human leukocyte elastase (1.0 µg mL^− 1^) was incubated for 20 min with HEPES buffer (4- (2-hydroxyethyl)-1-piperazine ethane sulfonic acid, pH 7.5) and different concentrations of EOs or elastase inhibitor (methoxy succinyl-alanyl-alanyl-prolyl-valine chloromethyl ketone (MeOSuc-AAPV-CMK), 1.4 mg/mL; Sigma, Aldrich, United States) in 96-well plate at room temperature then added 100 µL from the substrate (1.0 mM of N-methoxy succinyl - Ala - Ala - Pro -Val - *p* -nitroanilide, Sigma-Aldrich, United States). The absorbance was measured at λ_405_ compared to the blank after 40 min.

#### Estimation of anti-collagenase activity

Type 1 collagenase enzyme (1.0 mg mL^−^1) [was isolated from *Clostridium histolyticum* (ATCC 17859) obtained from the national research center Micro-lab, El-Giza, Egypt] and dissolved in 50.0 mM tricine buffer (pH 7.4) then incubated for 20 min at 37◦C with EOs different concentrations or 1.40 mg mL^− 1^ L EDTA (standard), then 100 µL of FALGPA substrate (N-(3-[2-Furyl] acryloyl)-Leu-Gly-Pro-Ala (Sigma, Aldrich, United States) was added. After further incubation for 60 min at 37◦C, 200 µL of ninhydrin (2%) in citrate buffer (pH 5.0, 200 mM) was added and placed in the water bath at 100 ◦C for 5 min, then cooled followed by the addition of 50% isopropanol (200 µL). The final absorbance was measured at λ_540_ nm.

#### Estimation of anti-tyrosinase activity

It was estimated by incubation of mushroom tyrosinase (5600 units mL^− 1^) with different concentrations of EOs or the standard kojic acid (1.4 mg mL^− 1^) for 15 min at 37◦C, then 1.0 mM of L-DOPA (substrate) was added. The absorbance was measured at λ_475_ nm.

In all three methods, a curve was plotted as a relation between sample concentration and % inhibition from which the IC_50_ was detected using non-linear regression analysis.

### Assessments of anti-bacterial activity

#### Material for anti-bacterial activity

Gram-positive bacteria stock cultures (*Streptococcus pyogenes* ATCC 12344, *Staphylococcus aureus* ATCC 25923, *Clostridium perfringens* ATCC 13124), and stock cultures of the Gram-negative *Pseudomonas aeruginosa* (ATCC 9027) were purchased from Institute of Research and Technology (Micro-lab), Vellore, Tamilnadu, India. The used microbiological media, Mueller-Hinton broth, Mueller-Hinton agar, as well as biological grade DMSO (sterile) and positive control antibiotics such as chloramphenicol (C), and gentamycin (CN) as 6.0 mm discs were obtained from Oxoid, Thermo Fisher Scientific (MA, USA).

#### Agar-well diffusion assay

It was performed following the Clinical and Laboratory Standards Institute (CLSI) and Gholizadeh et al. [37]. Each bacterial strain (100 µL, 1 × 10^5^ CFU/mL) was spread above the MHA medium, then 0.6 cm wells were done, and 50 µL of different EOs concentrations were added to each one. The plates were incubated after being kept in the refrigerator for 30 min at 37^o^C (24 h) for bacteria. The antimicrobial susceptibility of each sample was determined by measuring the developed zones of inhibition (ZOI) diameter in mm. The activity was compared to positive control antibiotics such as gentamycin (10 µg/mL) and chloramphenicol (30 µg/mL) as protein synthesis inhibitors. Sterile DMSO in a diluted conc. was also used as a vehicle or negative control.

#### Determination of minimum inhibitory concentration (MIC)

MICs were measured using broth microdilution assay against the selected skin bacterial strains [38]. Stock solution of each obtained EOs sample was prepared by solubilizing 100 µL/mL in 1 mL DMSO then serial dilution (1/10) using sterile Mueller Hinton broth (MHB) was prepared. Thereafter, in 96 microtiter plates, about 100 µL of sterile MHB was added to each well, while 150 µL of the diluted stock samples were added in the first column only. Two-fold dilution for each 1/10 diluted sample was accomplished by transferring 100 µL from the first to the 11th well. Consequently, 100 µL of each microbial inoculum (1 × 10^5^ CFU/mL) was transferred to each well except the last one, which was kept as a negative control. The microtiter plate was incubated at 37^o^C for 24 h after which the absorbances were measured at λ_max_ 620 nm using an automated microplate reader (ChroMate 4300, USA) and represented graphically using Microsoft Excel version 2019.

### Statistical analysis

In vitro antioxidant data was collected from three independent experiments. Values were stated as mean ± SD and the IC_50_ of each tested sample was calculated from the non-linear regression analysis from the curves plotted between log sample concentration and the measured absorbance or fluorescence implemented on GraphPad Prism version 5.0 (San Diego, CA, United States).

## Results and discussion

The selection of a suitable method for extracting volatile components from aromatic plants is a critical step [39]. For instance, the conventional HD extraction method is the most commonly used technique with affordable cost and simple setup [26], while the operating temperature may affect the quality of the extracted oil. On the other hand, the SF and HS extraction methods are express, environmentally friendly, and reserve the chemical nature of the extracted components [26]. Accordingly, in the present study, the EO of *M. rugulosa* leaves cultivated in Egypt was extracted using HD, SF, and HS for the first time to compare the impact of the extraction method and the applied condition on the chemical composition, hence the biological activities. Our result shows that the extraction technique affects the color, consistency, and yield of EOs. HD EO was noticed to be deep yellow with a nice odor and low viscosity, while SF extract was dark with a light pleasant odor and highly viscous consistency. Regarding the yield, SF extraction gave the largest yield (0.8%), which was three times more than that of HD (0.26%), while in the case of the HS method the oil was unrecoverable. To sum up, the yield and physical properties of the extracted EOs were almost consistent with the reported characters of each technique. For instance, the supercritical CO_2_ used in the SF extraction is non-viscous, has low surface tension with high diffusion power, and so gives a high oil yield [40]. HS technique is used mainly for quantifying the volatile constituents using the dynamic head space but with no yield [41].

### *GC/MS analysis of M. rugulosa leaves’ volatile constituents*

Keen interpretation of the GC/MS data revealed the identification of seven, eleven, and nine volatile components in HD, HS, and SF, representing 98.99%, 99.3%, and 54.75%, respectively (Table 1, Supplementary Figure S1–S3). Concerning the nature of the chemical class, the oxygenated components predominate in the three techniques, while the estimated percentage varies as per the applied method. The highest percentages were detected in HD (95.93%) and HS (80.94%), while the lowest was observed in the SF extract (48.4%), respectively. In the case of SF, the oxygenated monoterpene (OM) and sesquiterpene (OS) represent 18.81 and 13.32%, respectively. Monoterpene hydrocarbon volatiles showed the highest percentage in the HS aroma (18.43%). The rationale behind the discrepancy in the percentage and nature of the identified compounds may be correlated to the slightly polar and highly volatile nature of oxygenated compounds due to the presence of oxygenated pharmacophores. Accordingly, they can be easily extracted by hot water as in the HD method or by heating as in HS. On the other side, supercritical CO_2,_ used in the SF extraction method, has a non-polar solubility power, accordingly, it dissolves mainly non-polar substances (monoterpene hydrocarbons) with limited solvation to the polar, oxygenated constituents. An in-depth interpretation of the results revealed that eucalyptol (1, 8-cineol) represents the major identified component in the HD-EO and HS volatiles but with dissimilar percentages being 89.60% and 73.13%, respectively. On the other hand, *dl*-*α*-tocopherol and *α*-terpineol represent the major components in the SF extract, being 16.27% and 11.89%, respectively. Interestingly, the identified components in the HD-EO results were in coincided with the formerly reported data [23, 27], however, they were dissimilar in the calculated percentage which may be, due to the environmental conditions including season of collection and geographical impact [42].

**Table 1:** Average percent concentration (%) of the volatile components identified in M. rugulosa leaves extracted using HD, HS and SF

No.	Compound	Chemical class	MF	M. wt.	RI_Exp_^[a]^	RI_Lit_^[b]^	HD	HS	SF
							Average % Conc. (*n* = 3)		
1.	2-Hexenal-E	OM	C_6_H_14_O	98.14	822	833	------	3.97	------
2.	Isoamyl acetate	OM	C_7_H_14_O_2_	130.187	859	860	-----	0.36	------
3.	*α*-Pinene	MH	C_10_H_16_	136.23	948	948	1.28	5.34	------
4.	*β*-Pinene	MH	C_10_H_16_	136.23	943	947	0.72	2.01	------
5.	*O*-Cymene	MH	C_10_H_14_	134.22	1042	1041	0.52	------	------
6.	Eucalyptol	OM	C_10_H_18_O	154.25	1059	1053	**89.60**	**73.13**	5.50
7.	D-Limonene	MH	C_10_H_16_	136.23	1025	1025	-----	**7.46**	------
8.	*α*-Terpineol	OM	C_10_H_18_O	154.25	1143	1143	4.85	2.6	**11.89**
9.	Caryophyllene	SH	C_15_H_24_	204.35	1444	1444	------	------	3.74
10.	Aromadendrene	SH	C_15_H_24_	204.35	1452	1452	------	------	0.95
11.	Germacrene B	SH	C_15_H_24_	204.35	1488	1549	------	------	1.66
12.	(-)-Spathulenol	OS	C_15_H_24_O	220.35	1569	1572	------	------	6.54
13.	Viridiflorol	OS	C_15_H_26_O	222.37	1575	1573	------	------	6.78
14.	dl-*α*-Tocopherol		C_29_H_50_O_2_	416.7	3149	3149	------	------	**16.27**
15.	*β*-Myrcene	MH	C_10_H_16_	136.24	958	957	0.54	2.57	------
16.	*β.*-Cymene	MH	C_10_H_14_	134.22	1042	1037	------	0.66	------
17.	γ-Terpinene	MH	C_10_H_16_	136.23	1053	1053	------	0.39	------
18.	Terpinen-4-ol	OM	C_10_H_18_O	154.25	1137	1140	1.48	0.88	1.42
**Total identified compounds**							98.99%	99.3%	54.75%
**Non-oxygenated monoterpene hydrocarbons (MH)**							4.54%	18.43%	6.35
Monoterpene hydrocarbons (MH)							4.54%	18.43%	6.35
Sesquiterpene hydrocarbon SH))							-	-	-
**Oxygenated compounds**							95.93%	80.94	48.4
Oxygenated monoterpene (OM)							95.93%	76.61%,	18.81%
Oxygenated sesquiterpene (OS)							-	-	13.32%

MF Molecular formula, M. wt.: Molecular weight, RI_Exp_: experimental retention index, RI_Lit_: reference retention index; HD: hydro distillation, HS; head space; SF: supercritical fluid

### Antioxidant, antiaging, and anti-hyperpigmentation activity of extracted volatiles from M. Rugulosa leaves

Aging progression can be triggered by endogenous or exogenous factors that are greatly linked with oxidative stress, *via* the formation of reactive oxygen species (ROS) [43]. ROS directly damages skin cells, mediates inflammatory responses, and enhances the degradation of the vital extracellular matrix components. Therefore, topical application of antioxidant agents can be valuable in inhibiting molecular damage and sustaining skin homeostasis. Among other antioxidants, EOs have recently gained recognition as therapeutics for well-being care. Their topical application is relatively safe, and adverse events are minor, self-limiting, and occasional. For instance, Tea Tree oil, a well-known EO extracted from Melaleuca species, is reported to be toxic if ingested in higher doses or cause skin irritation if topically applied at higher concentrations, while noticed for being safe if diluted or applied at low concentrations [44]. In this regard, herein we evaluate the antioxidant activity of *M. rugulosa* HD-EO and SF extract using different in vitro enzyme-based techniques to assess their antioxidant mechanism [45, 46]. For instance, the DPPH radical scavenging, and oxygen radical absorbance capacity assays depend on the electron transfer mechanism, while the *β*-carotene bleaching assay is based on hydrogen atom transfer [47]. Our results (Table 2) showed that HD-EO and SF extract exhibit far DPPH radical scavenging capacity with IC_50_ 16.6 ± 3.65 and 13.0 ± 2.75 µL/mL, respectively, in comparison to the standard (ascorbic acid, IC_50_ 1.83 µg/mL). On the other side, they displayed potent radical scavenging properties in the ORAC assay with IC_50_ 16.9 ± 4.12 and 12.0 ± 0.89 µL/mL for HD and SF samples, respectively which is more effective than the standard drug (Trolox: 27.0 ± 13.41 µg/mL). Finally, the results of the *β*-carotene bleaching assay revealed that the HD-EO possessed IC_50_ 7.51 ± 0.83µL/mL which is comparable to the inhibitory activity of the standard BHT (IC_50_ 8.06 ± 0.67 µg/mL). Meanwhile, the SF extract showed IC_50_ = 6.91 ± 0.15µL/mL which is even more effective than the standard (BHT). In all, the tested EOs showed promising antioxidant capability in all assays except the DPPH radical scavenging test which may be due, at least in part, to the low miscibility of the EO in the test reagents [48]. The GC/MS analysis of the HD-EO pinpointed 1,8-cineole as the leading oil component (89.60%). In prior studies, 1,8-cineol has established potent free radical scavenging activity in different in vitro assays, highlighting its ability to neutralize ROS, protect against oxidative damage to the cell, and enhance cellular defense mechanisms [49]. Accumulation of ROS results in oxidative stress which is involved in the emerging and progression of different pathological disorders, just as inflammation and aging [50]. Moreover, the antioxidant ability of 1,8-cineole extends beyond its capacity to direct ROS scavenging as it can chelate metal ions [51]. Sequestering these ions can limit the formation of ROS and protect against oxidative damage. Although 1,8-cineole is established as the main volatile component in the HD EO and plays a fundamental role in the activity, the significance of minor volatiles on the bioactivity is stated in many studies [52]. Herein, *α*-terpineol, *α*-pinene, and *β*-myrcene were estimated in the HD-EO in a low percentage, however, they possess considerable radical scavenging and potent ORAC activity [53, 54]. On the same approach, the antioxidant activity of the SF extract may be attributed, at least in part, to dl-*α*-tocopherol content (16.27%) which is a very active antioxidant agent, acts as a free radical and singlet state oxygen scavenger, hence playing an essential role in skin protection from free-radical-generating agents [55]. Other antioxidants detected in SF extract in moderate to low concentrations are *α*-terpineol [53], caryophyllene [56], and spathulenol [57]. Although SF extract is rich in dl-*α*-tocopherol content with a lower percentage of 1,8-cineole and the HD-EO is chiefly prominent with 1,8-cineol, SF displayed better antioxidant potential than HD-EO. This could be rationalized by the fact that EOs composed of major and several minor volatile constituents, the minor compounds play a vital role in the activity through a synergistic or additive effect with the major components [28, 58–60]. In addition, tocopherol is renowned in the literature as a more powerful antioxidant than 1,8-cineol. For instance, in the DPPH assay, 1,8 cineol recorded IC_50_ of 63.79 µg/mL [61] compared to 13.4 µg/mL for tocopherol [62].

**Table 2:** Antioxidant activity of the *M. rugulosa* leaves volatile components extracted by HD and SF

Tested samples	IC_50_ ± SD (µL/mL)		
	DPPH	ORAC	β-Carotene
HD	16.6 ± 3.65	16.9 ± 4.12	7.51 ± 0.83
SF	13.0 ± 2.75	12.0 ± 0.89	6.91 ± 0.15
Ascorbic acid*	1.83 ± 1.41	-	-
Trolox*	-	27.0 ± 13.41	-
BHT*	-	*-*	8.06 ± 0.67

*µg/mL; HD: hydro distillation, HS; head space; SF: supercritical fluid

Subsequently, the extracted volatile components, either by HD or SF were screened for their in vitro antiaging and anti-hyperpigmentation activity through inhibition of elastase, collagenase, and tyrosinase enzymes. Our results, (Table 3**)** displayed that the HD-EO exhibited potent anti-tyrosinase activity with IC_50_ 285.87 ± 1.89 µg/mL [inhibition percentage, (IP) 85.17 ± 1.77] compared to IC_50_ 362.5 ± 2.08 µg/mL for SF extract (IP, 65 ± 1.63) and the stander Kojic acid (321.65 ± 3.41 µg/mL). These results follow previously published data that the EO with the relatively low-oxygenated terpenoids displayed better tyrosinase inhibitory activity [63]. EOs containing hydrophobic compounds acted as competitive inhibitors on the tyrosinase, hence on melanin synthesis. Tyrosinase is considered the key enzyme in the biosynthesis of melanin and dermatological disorders that result from the extreme accumulation of melanin. So, tyrosinase inhibitors have become increasingly important for treating skin disorders and are valuable therapeutics for skin hyperpigmentation [64]. Additionally, tyrosinase is a copper-containing enzyme so antioxidants with metal chelation properties are considered an effective tyrosinase inhibitor [65]. Reportedly, *Melaleuca* EOs are rich in cineol and exhibit significant reducing power [66]. Therefore, the expected inhibition mechanism for tyrosinase might be through the chelation of copper ion of the tyrosinase enzyme and destruction of tautomerization to dopachrome, thus EOs act as reducing agents on melanin intermediates by blocking oxidation chain reaction at various points from tyrosinase/ DOPA to melanin and hence causing reduction of skin pigmentation [67].

**Table 3:** The inhibitory effect of *M. rugulosa* leaves volatile components extracted by HD, and SF on the elastase, collagenase, and tyrosinase enzyme

Tested samples	IC_50_ ± SD (inhibition percentage (IP) ± SD)		
	Anti-elastase	Anti-collagenase	Anti-tyrosinase
HD	62.56 ± 2.32 (81.18 ± 1.45)	390.17 ± 1.71 (58.65 ± 1.31)	285.87 ± 1.89 (85.17 ± 1.77)
SF	57.2 ± 2.88 (88.0 ± 1.35)	335.34 ± 2.09 (65.05 ± 1.48)	362.5 ± 2.08 (65 ± 1.63)
MeOSu-AAPVCMK*	44.92 ± 1.71 (92.52%± 4.63)	-	-
EDTA*	-	315.12 ± 2.21 (79.82%± 2.63)	-
Kojic acid*		-	321.65 ± 3.41 (76.52%± 0.83)

*Conc. µg/mL; HD: hydro distillation; SF: supercritical fluid

Moreover, the SF extract (IC_50_ 57.2 ± 2.88 µg/mL) and HD-EO (IC_50_ 62.56 ± 2.32 µg/mL) showed strong anti-elastase activity with IP 88 ± 1.35 and 81.18 ± 1.45 for SF and HD, respectively comparing to the anti-elastase standard (IC_50_ 44.92 ± 1.71 µg/mL, IP 92.52% ± 4.63). Additionally, the SF sample showed better anti-collagenase activity with IC_50_ 335.34 ± 2.09 µg/mL (IP, 65.05 ± 1.48) than that of HD IC_50_ 390.17 ± 1.71 (IP 58.65 ± 1.31) in comparison to EDTA (315.12 ± 2.21 (IP 79.82%± 2.63). Skin proteins such as collagen and elastin are essential in preserving the skin’s healthy appearance and integrity [68]. Collagen, a leading protein in the cutaneous extracellular matrix (ECM), is responsible for strengthening the skin, while under certain conditions its triple helical structure was cleaved at the terminal amino acid-glycine bond by collagenase enzyme (a metalloprotease enzyme) causing skin aging [69]. Another proteolytic system involved in the degradation of the ECM is that of serine proteases, one of which is elastase. Elastase, a member of the chymotrypsin family of proteases, is responsible primarily for the breakdown of elastin, an important protein found within the ECM. Elastin has unique elastic recoil properties and is vital for giving elasticity to the skin [70]. Elastases can cleave elastin with a broad substrate portfolio including the ability to hydrolyze collagen and other ECM proteins with subsequent skin sagging and wrinkling [70]. It is noteworthy that exposure to UV radiation, and free radicals are among the factors that spark the activation of both enzymes with subsequent destruction of collagen and elastin, loss of skin elasticity, and the appearance of wrinkles [71]. Therefore, finding inhibitors for elastase and collagenase is an effective strategy to protect the skin from aging manifestations. In this context, our results mirrored significant anti-aging activities correlated to the antioxidant potential of the identified volatile components. Volatile constituents in our investigated extracts such as 1,8-cineol, *α*-terpineol, tocopherol, and spathulenol might be involved in enzymatic inhibition by multiple mechanisms. Firstly, the hydroxyl group in the core structure of the identified antioxidants could interact with critical functional groups in the backbone or side chain of collagenase and elastase, altering their proteolysis function [26]. Secondly, the hydrophobic interaction between the antioxidant and active sites causes conformational changes, hence enzyme inhibition [72]. Thirdly, the metal chelator capacity of antioxidants promotes their interaction with the Zn ion active site and prevents the enzymes from exerting their proteolytic action [73]. Ultimately, our promising antiaging results were on the same approach as reported literature on other melaleuca species in that all possess significant anti-collagenase and anti-elastase activity [26, 74].

### In vitro antibacterial activity

The antimicrobial activity of the *M. rugulosa* EOs was evaluated against four common skin pathogens namely, *P. aeruginosa* (Gram-negative), S. *aureus*,* S. pyogenes*, and *C. perfringens* (Gram-positive). The results were obtained by measuring the developed zone of inhibitions (ZOI) in mm in the agar well-diffusion test and evaluating the MICs in the broth microdilution assay.

#### Agar-well diffusion assay

The results represented in Table 4, Supplementary Figures S4-S5, explicitly show the susceptibility of *S. aureus ATCC* to the HD EOs with more or less the same ZOI (12–19 mm), while *C. perfringens* ATCC 13124 was susceptible to SF-extract. The remaining bacterial strains are not sensitive to the tested EOs. The inhibitory effect strength was compared to chloramphenicol and gentamycin antibiotic discs as positive controls.

**Table 4:** Zones of inhibition of HD and SF-extracted volatile components from *M. rugulosa* leaves against reference skin-related bacterial pathogens in comparison to standard antibiotics using agar well diffusion assay

Reference strains	EOs (µL/mL)						Antibiotics (µg/mL)	
	HD			SF			C	CN
	5	10	20	5	10	20	30	10
	ZOI in mm							
*Gram-positive bacteria*								
*S. aureus (ATCC 25923)*	12	16	19	Nz	Nz	Nz	13	-
*S. pyogenes (ATCC 12344)*	Nz	Nz	Nz	Nz	Nz	Nz	9	10
*C. perfringens (ATCC 13124)*	Nz	Nz	Nz	13	14	16	18	12
*Gram-negative bacteria*								
*P. aeruginosa (ATCC 9027)*	Nz	Nz	Nz	Nz	Nz	Nz	9	-
10% DMSO (-ve control)	Nz	Nz	Nz	Nz	Nz	Nz	-	-

C: Chloramphenicol, CN: Gentamycin, Nz: No zone of inhibition was observed

### Broth microdilution assay

The MIC results (Table 5) showed the promising ability of the tested HD EO to suppress the growth of the selected skin-related microbial strains *S. aureus* with (MIC = 5.0 µg/mL), while, the SF-extract, displayed inhibitory activity against *C. perfringens* with MIC = 20.0 µL/mL. Concerning the significance of the extracted EOs in skin infection, the antibacterial activity of the HD-EO and SF-extract was evaluated against four common skin pathogens, namely, *P. aeruginosa*, *S. aureus*, *S. pyogenes*, and *C. perfringens*. They were nominated depending on their availability and pathogenic history of skin infection. For instance, *S. aureus* is a commensal pathogen often present on the skin and mucous membranes of healthy individuals [75] and it is responsible for 80–90% of all skin and soft tissue infections in humans throughout the world [76–78], as well as, it easily acquires antimicrobial resistance through mutation or horizontal transfer of resistance genes from other bacteria. *S. pyogenes* (group A Streptococcus) is one of the most important pathogenic bacteria that cause skin and soft tissue infections worldwide. It can cause infection in the superficial keratin layer, the superficial epidermis, the subcutaneous tissue, and the fascia. It is also the etiologic agent of scarlet fever and streptococcal toxic shock syndrome [79, 80]. *P. aeruginosa* is one of the most common pathogens responsible for infections in hospitalized patients. It is commonly associated with wounds, burns, bedsores, abscesses, and other superficial infections [81]. *C. perfringens* is responsible for tissue necrosis, bacteremia, emphysematous cholecystitis, and gas gangrene [82, 83]. Our results revealed the sensitivity of *S*. *aureus* to the tested HD-EO and the susceptibility of *C. perfringens* to the SF-extract. The antimicrobial activity of the EOs is highly correlated, at least in part, to the chemical constituents in each oil. The HD-EO is rich in 1,8-cineol as a cyclic oxygenated monoterpene and possesses a potent anti-staphylococcal effect [84, 85]. Furthermore, it was reported that 1,8-cineol was active against a wide range of pathogenic strains with various MICs [86] due to its small size and non-polar structure. Moreover, it affects the permeability of the bacterial cell and membrane fluidity leading to a change in the topology of membrane proteins and stopping the respiratory process of the cell [87, 88]. Moreover, the antibacterial mechanism is linked with ROS in the cells, which induce oxidative stress and consequently inhibit certain essential biological processes [89, 90]. Additionally, the SF sample contains a large percentage of *α*-terpineol. Reportedly, EO rich in *α*-terpineol showed antibacterial activity against *C. perfringens*. Moreover, the SF extract contains a high percentage of *dl-α*-tocopherol and possesses promising antimicrobial activity [91]. Tintino and co-workers [92] reported that the potential antibacterial mechanism of tocopherol may be due to its lipophilic character which causes loss of integrity and malfunction in the efflux pumps and membrane transporter [92]. However, it is important to note that the mentioned properties may be due to synergistic interaction between minor and major compounds.

**Table 5:** MICs of the HD and SF-extracted volatile components from *M. rugulosa* leaves against reference skin-related Gram-positive pathogens using a broth microdilution assay

Susceptible organisms	MIC (µL/mL)	
	HD	SF
*S. aureus (ATCC 25923)*	5	-
*S. pyogenes (ATCC 12344)*	-	-
*C. perfringens (ATCC 13124)*	-	20

### Metabolomics studies

The GC/MS data was processed by MZ mine 2.53. according to a workflow designed previously [93] and identified by the NIST database for dereplication purposes. Principle component analysis (PCA) was carried out to test the similarities and differences in the chemical profile of the tested extracted essential oils.

**Fig. 1:**
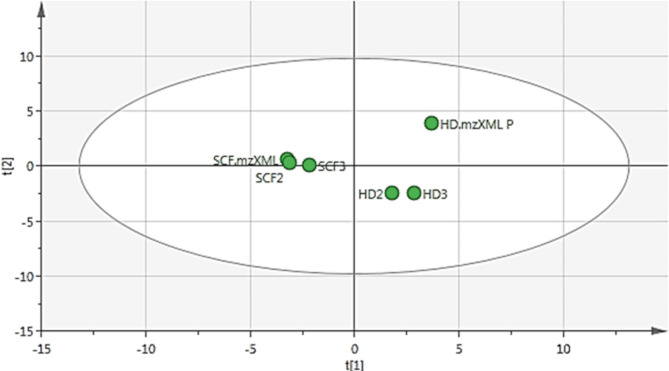
PCA score plot

**Fig. 2:**
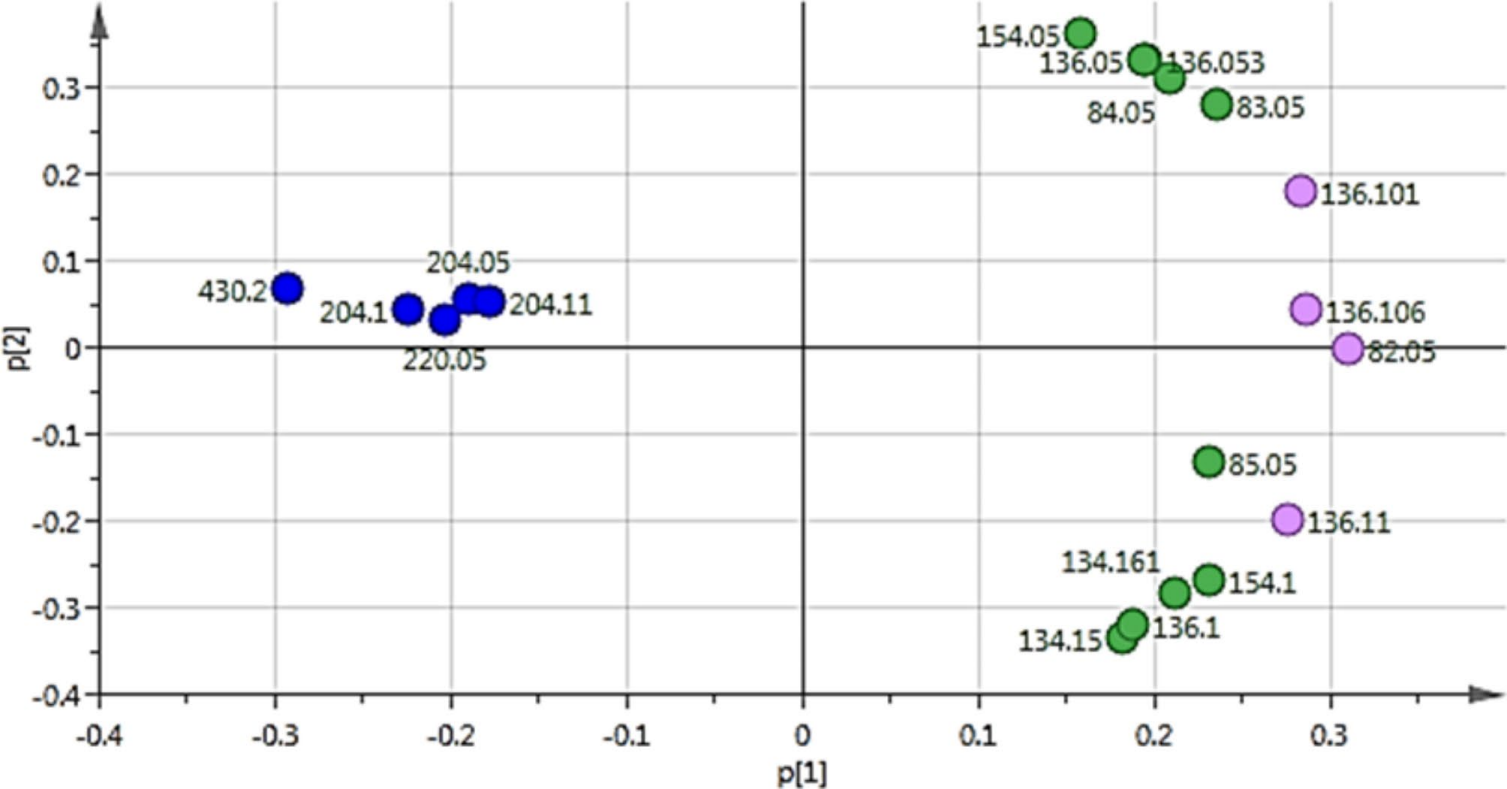
PCA loading plot

**Fig. 3:**
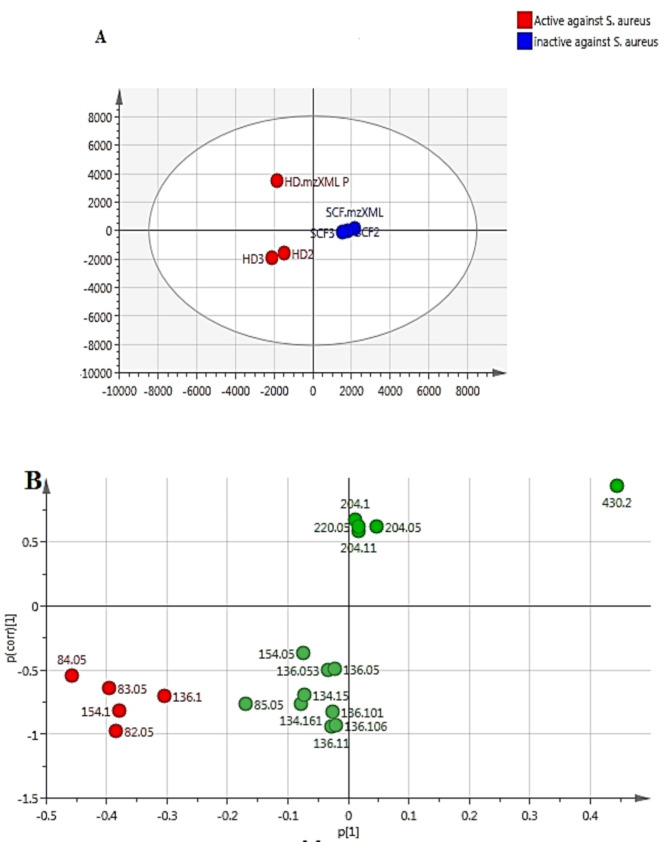
(**A**) Orthogonal partial least squares discriminant analysis (OPLS-DA) score plot of the active HD EOs as antibacterial against *S. aureus* strain (red circles) versus inactive SF-extracts (blue circle). (**B**) S-Loading plot showing the putatively active metabolites for antibacterial activity against the *S. aureus* strain

PCA is an unsupervised multivariate data analysis that shows clusters, groups, and outliers among the observations [94]. PCA score plot (Fig. 1) with quality of goodness R2 = 0.822 and model predictability Q2 = 0.589 referring to a strong fit model with high predictive power. PCA loading plot (Fig. 2) highlighted the metabolites that contributed to the variations among the different observations, as SF-extract was characterized by molecules at *m/z* (retention time in minutes) 430.2 (R_t_ 53.32) corresponding to dl-*α*-tocopherol and 204.1 (R_t_ 22.9 and 23.8) corresponding to caryophyllene and aromadendrene, respectively. Moreover, HD-EO was distinguished by the outliers at *m/z* 136.1 (R_*t*_ 8.6, 9.1, and 11.17) corresponding to *β*-pinene, *β*-myrcene, and *γ*-terpinene. Orthogonal partial least square discriminant analysis (OPLS-DA) was applied to highlight the molecules highly correlated with the antimicrobial activity. OPLS-DA score plot (Fig. 3) showed a clear discrimination between the active antimicrobial HD against *S. aureus* and the other inactive SF extract. The model’s measures for goodness of fit R2 = 0.949 and prediction Q2 = 0.955. The molecules highly correlated with HD EO antimicrobial activity against *S. aureus* were checked with high coefficients of variation. The descriptive statistics of the model led to the identification of significant elements at m/z 154.1 (R_t_ 10.38) identified as eucalyptol which represents the major compound, in addition to 136.1 (R_t_ 10.4) identified as D-limonene. Moreover, The OPLS-DA score plot (Fig. 3A) demonstrated complete discrimination between the active SF-extract against *C. perfringens* and the inactive HD EO. The molecules highly correlated with SF-extract antimicrobial activity against *C. perfringens* were revealed in the S-plot (Fig. 3B). The S-plot showed significant elements at *m/z* 430.2 (R_t_ 53.34) identified as *dl-α*-tocopherol which was reported for its antimicrobial activity [91].

## Conclusion

The chemical profile and yield of volatile components extracted from *M. rugulosa* leaves cultivated in Egypt, displayed significant variations concurrently with the implemented extraction method. Oxygenated volatiles are dominant in the HD-EO (95.93%), and HS aroma (80.94%), while the SF extract displayed the least content (48.4%). Eucalyptol (1,8-cineol) is the key component in the HD (89.60%) followed by HS (73.13%), while *dl*-*α*-tocopherol and *α*-terpineol represent the highest percentage in SF comprising 16.27% and 11.89%, respectively. The chemical fingerprint of the different extracts was further estimated using multivariate data analysis, where eucalyptol and tocopherol represented the major phyto-markers contributing to their segregation. Regarding the bioactivity profile, HD-EO and SF extract possessed antioxidant, anti-elastase, anti-tyrosinase, and anti-collagenase potential compared to reference standards. The HD-EO shows selective antibacterial against the gram-positive *S. aureus*. OPLS-DA and dereplication studies predicted the antibacterial against Gram-positive, *S. aureus* strain (MIC 5 µL/mL) whereas, SF extract showed significant antibacterial activity against the resistant Gram-negative bacteria, *C. perfringens* (MIC 20 µL/mL). OPLS-DA predicted that this activity was related to major volatile constituents in each sample, we can’t neglect the synergistic effect of minor volatiles and the potency of each single component. To sum up, *M. rugulosa* volatiles extracts could be promoted as promising therapeutics for the control of skin aging and infection, however, further toxicity and clinical investigation is recommended.

## Electronic supplementary material

Below is the link to the electronic supplementary material.

Supplementary Material 1

## Data Availability

All data generated or analyzed during this study are included in the manuscript and/or its supplementary information files.

## Declarations

### Experimental research and field studies on plants

We confirm that all methods were performed by the relevant guidelines and regulations, and comply with relevant institutional, national, and international guidelines and legislation.

## Abbreviations

BHT: Butylhydroxytoluene
DPPH: 2,2-Diphenyl-1-picrylhydrazyl radical
EO: Essential oil
GC/MS: Gas Chromatography/Mass spectrometry
HD: Hydrodistillation
HS: Headspace
MIC: Minimum inhibitory concentration
ORAC: Oxygen radical absorbance capacity
OPLS-DA: Orthogonal partial least square discriminant analysis
PCA: Principal component analysis
R2: Quality of goodness
ROS: Reactive oxygen species
SF: Supercritical fluid
Q2: model predictability
ZOI: Zone of inhibition
