# Supplementary material for: Volatiles extracted from Melaleuca Rugulosa (Link) Craven leaves: comparative profiling, bioactivity screening, and metabolomic analysis
Source: BMC Complement Med Ther. 2024 Nov 13;24:394. doi: 10.1186/s12906-024-04683-z (PMC11562704; doi:10.1186/s12906-024-04683-z)
Supplement: Supplementary file 1 — Supplementary Material 1 [file 12906_2024_4683_MOESM1_ESM.docx]

**Supplementary data**

**
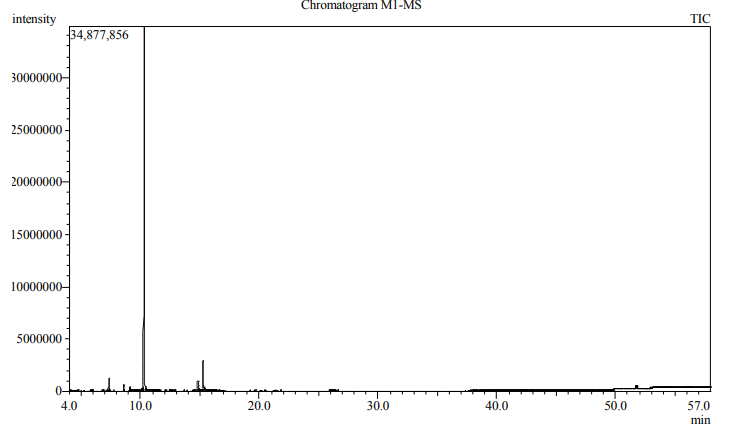
**

**Figure S1 A: Total ion chromatogram for the essential oil obtained by hydrodistillation (HD) extraction**

**
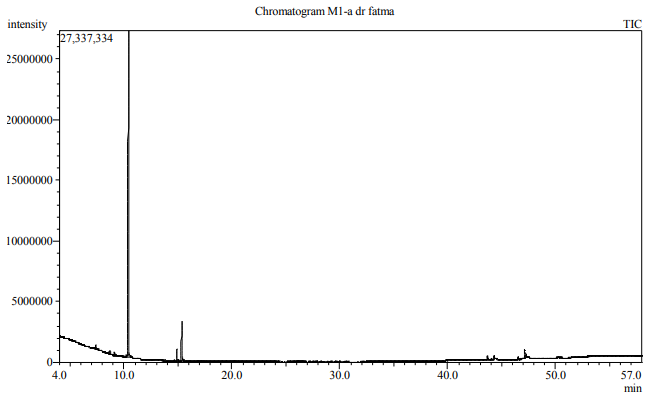
**

**Figure S1 B: Total ion chromatogram for the essential oil obtained by hydrodistillation (HD) extraction**

**
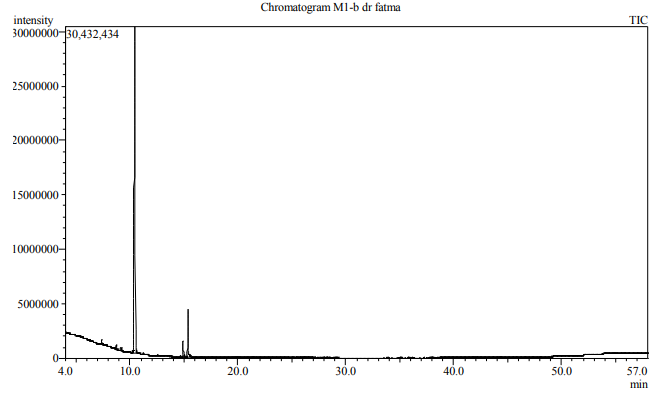
**

**Figure S1 C: Total ion chromatogram for the essential oil obtained by hydrodistillation (HD) extraction**

**
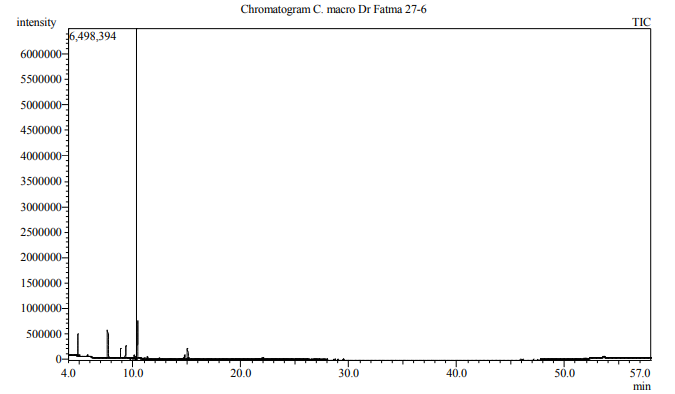
**

**Figure S1 A: Total ion chromatogram for the essential oil obtained by head space (HS) microextraction**

**
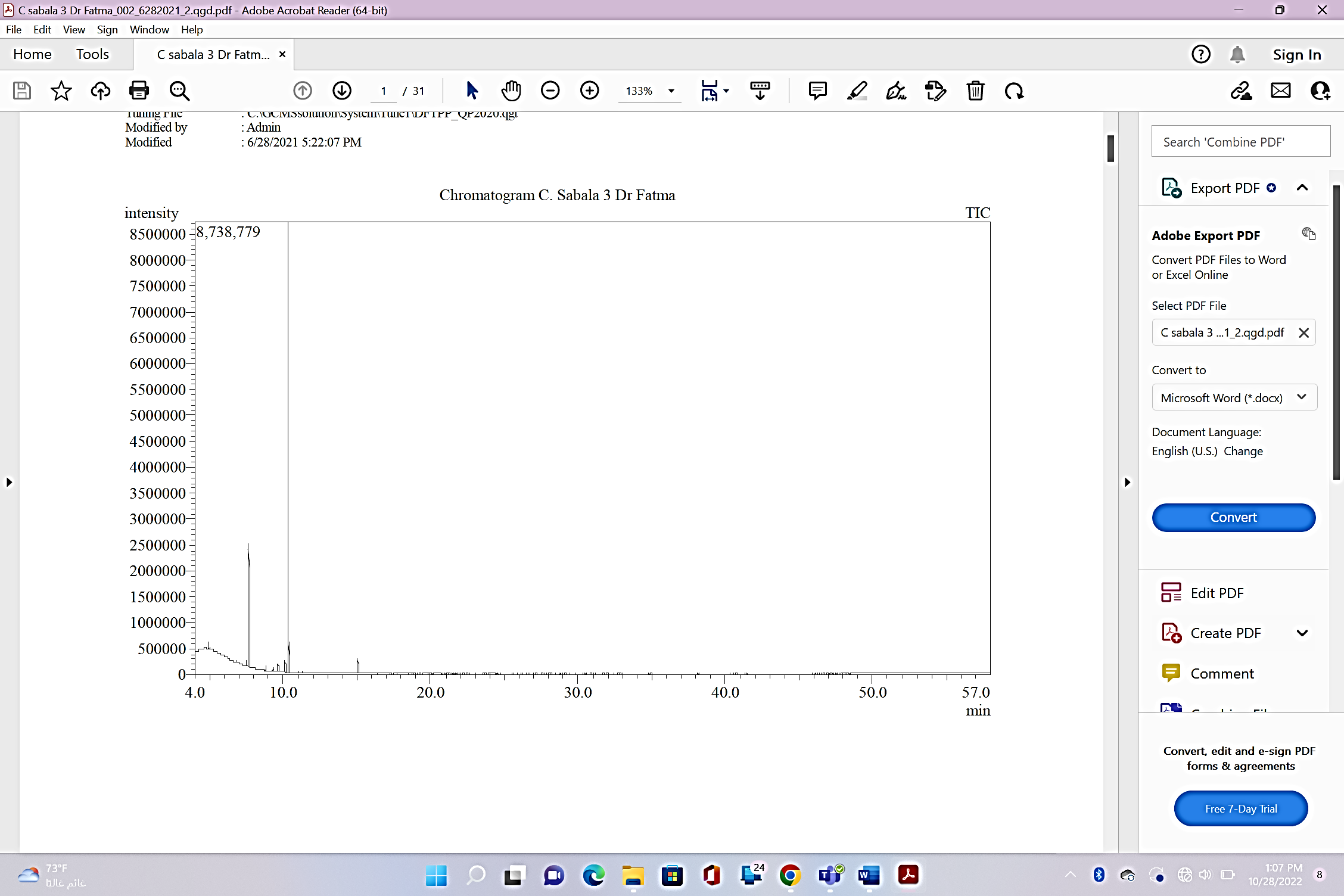
**

**Figure S1 B: Total ion chromatogram for the essential oil obtained by head space (HS) microextraction**

**
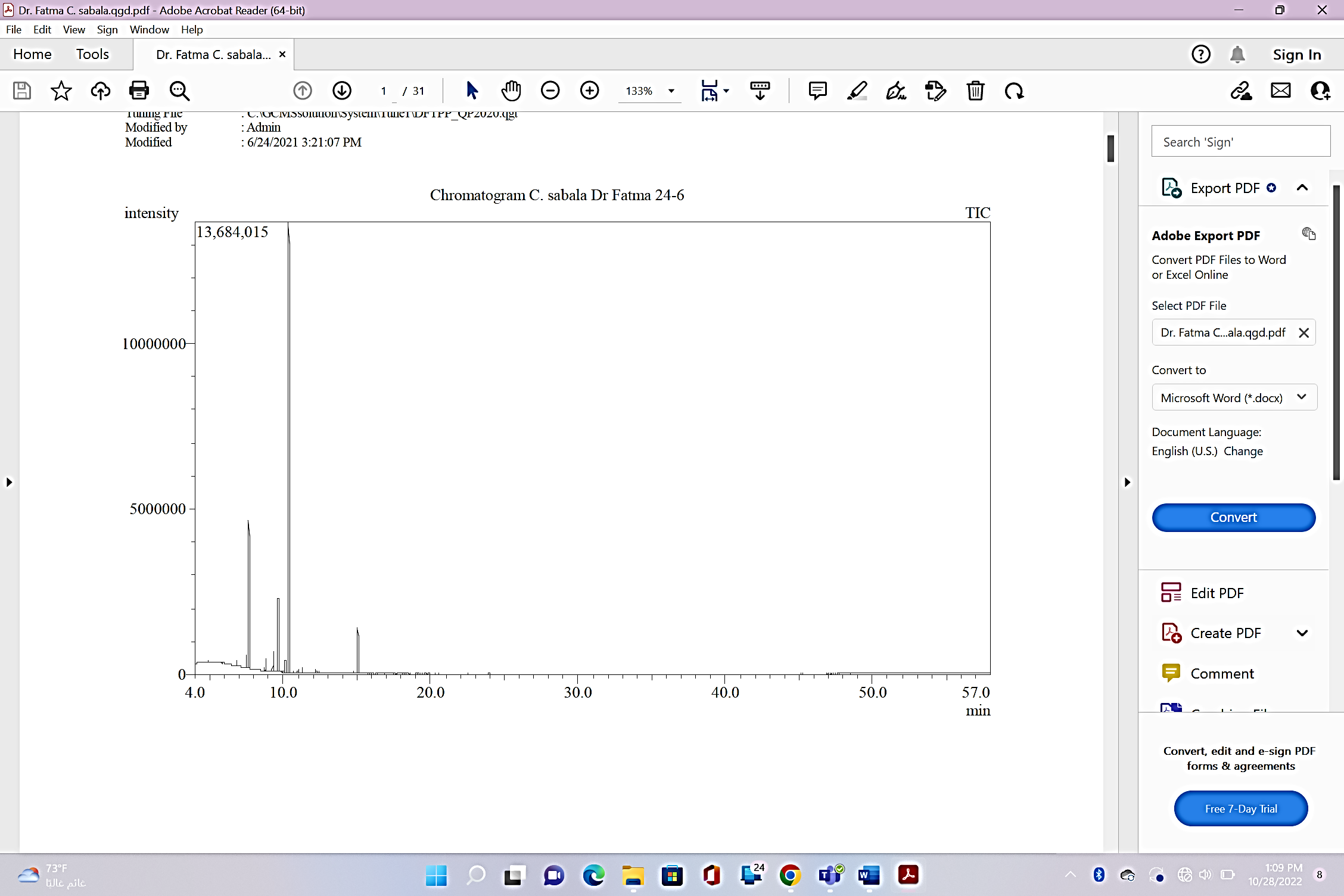
**

**Figure S1 C: Total ion chromatogram for the essential oil obtained by head space (HS) microextraction**

**
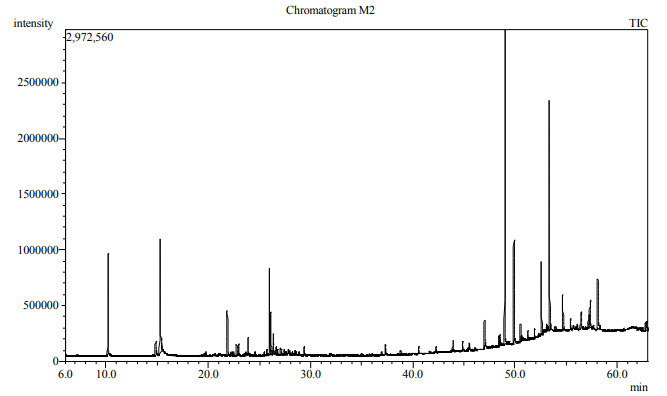
**

**Figure S3 A: Total ion chromatogram for the essential oil obtained by supercritical fluid (SF) extraction**

**
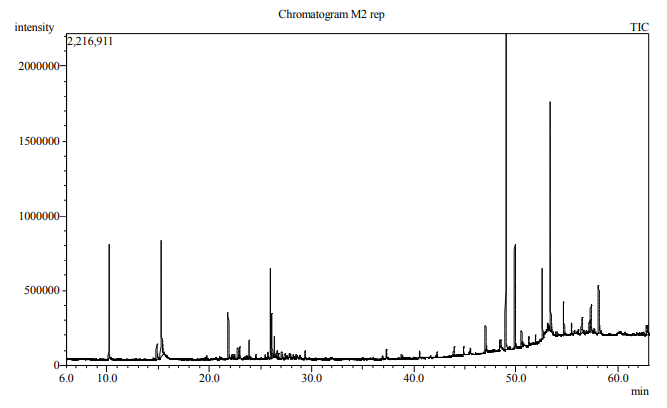
**

**Figure S3 B: Total ion chromatogram for the essential oil obtained by supercritical fluid (SF) extraction**

**
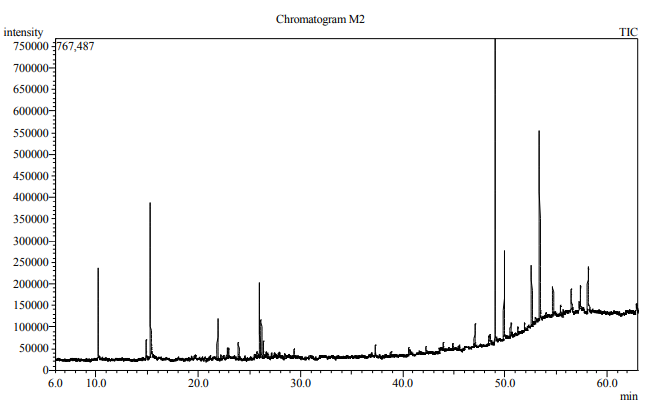
**

**Figure S3 C: Total ion chromatogram for the essential oil obtained by supercritical fluid (SF) extraction**

**Figure S4. Dose-response curve of the antimicrobial effect of** ***C. subulatus* EOs obtained by conventional HD method in broth microdilution assay.**

**Figure S5. Dose-response curve of the antimicrobial effect of *C.subulatus* EOs obtained by SF extraction method in broth microdilution assay.**
